# Supplementary material for: Are carbon emissions trading and green financial instruments synergistic? -Comprehensive quantitative research based on content analysis
Source: PLoS One. 2024 Mar 7;19(3):e0298601. doi: 10.1371/journal.pone.0298601 (PMC10919601; doi:10.1371/journal.pone.0298601)
Supplement: S1 Data — (ZIP) [file pone.0298601.s001.zip › Data/地级市Z_F工作报告.zip!/╔┬╬≈/═¡┤¿╩╨/└┤╫╘╣1⁄2╓┌║┼í╛┬φ┐╦╩2╛▌═°í┐_2018.docx]

各位代表：

　　现在，我代表市人民政府向大会报告工作，请予审议，并请市政协委员提出意见。

　　一、2017年工作回顾

　　过去一年，在省委、省政府和市委的坚强领导下，全市上下认真贯彻落实党的十九大和省十三次、市十二次党代会精神，自觉践行新发展理念，全面落实“五个扎实”要求和“五新”战略任务，聚焦追赶超越、转型发展主题，突出“四轮驱动”，强化“三区支撑”，有效应对各种困难和挑战，全市经济保持稳中有进、稳中向好态势，较好地完成了市十六届人大一次会议确定的目标任务。实现生产总值348.59亿元，增长7.6%，较上年加快0.6个百分点；完成地方财政收入20.06亿元，同口径增长9.3%；城乡居民人均可支配收入分别达到29928元、10346元，增长8.5%和9.2%。

　　（一）聚焦追赶超越，多措并举稳增长。面对多重困难叠加、下行压力加大的严峻形势，把稳增长作为首要任务，精准施策稳工业、促投资、强消费。先后出台了推进“五新”战略任务工作方案、建设西部传统产业转型升级示范城市行动方案、工业促投资稳增长的意见等一系列政策措施，扭转了经济增速放缓态势。全年新增规模以上工业企业28户，规模以上工业增加值增长7.5%，较上年加快1.5个百分点。项目建设常抓不懈，涉及项目377个、总投资2554亿元的重大项目库和重大项目综合管理平台上线运行，举行了3次重点项目集中开工活动，开展了项目观摩活动，120个市级重点项目完成投资220亿元，超额完成年度投资任务。全市固定资产投资完成476.29亿元，增长12.5%。招商引资签约项目260个，实际到位资金195.7亿元。新增限额以上商贸企业13户，社会消费品零售总额增长13.5%。

　　（二）紧扣转型发展，靶向施策调结构。出台了支持航汽铝材料工业、加快陶瓷产业发展等意见，建立了产业发展联席会议制度，航空汽车材料、装配式建筑、光伏发电等新兴产业发展势头良好。加快推进全域旅游、全景铜川建设，成功举办了第四届中国孙思邈中医药文化节暨第九届世界养生大会，50个旅游重点项目完成投资19.4亿元，游客接待量、旅游总收入分别增长25.5%和37.2%。服务业增加值占比41.4%，较上年提高1个百分点。我市成功入选国家中医药健康旅游示范区创建单位，节能减排财政政策综合示范工作实现“三连优”。

　　（三）夯实三农基础，聚力攻坚抓脱贫。坚持把脱贫攻坚作为重大政治任务和一号民生工程，扎实开展“冲在一线、干在实处，帮贫解困促发展”行动，精准实施各项脱贫工程。全市选派科技特派员208名，实现了科技特派员、专业合作社、互助资金协会、电商服务站贫困村全覆盖，荣获全省“万企帮万村”精准扶贫行动先进市。移民（脱贫）搬迁集中安置点竣工30个，建成安置房2058套，1642户5002名贫困群众喜迁新居。盐铜扶贫协作扎实推进。2625户9028名贫困人口实现脱贫，44个贫困村退出，超额完成年度减贫任务。全年粮食总产24.29万吨，水果总产79.16万吨。现代农业园区达到48个，汇聚各类龙头企业60家。在第24届杨凌农高会上，我市获得后稷特别奖16项、后稷奖17项，获奖数量居全省第一。

　　（四）坚持改革开放，创新机制增动能。全年完成各类改革任务108项。实施供给侧结构性改革“去杠杆”行动计划，国有企业“三供一业”分离移交工作顺利推进。商品房销售面积64.1万平方米。8户企业在陕西股权交易中心交易板挂牌。在全国地级市中率先开展普惠金融第三方评估，在全省率先选派金融系统优秀干部在重点镇挂职。“放管服”改革深入推进，“双随机、一公开”制度全面实施。制定了促进民营经济和中小微企业发展的意见，取消、停征或降低涉企行政事业性收费11项，全社会创新创业热情进一步激发，新增市场主体7383户，较上年增长20.4%；非公经济占比52.1%，较上年提高0.5个百分点。对外开放取得新进展，与意大利科内利亚诺市、尼日利亚克里斯河州建立了友好关系，与韩国奉化郡的友城关系不断深化。全年外贸进出口总额3.43亿元。

　　（五）统筹城乡发展，系统发力优环境。国家卫生城市通过复审，创建省级高新技术产业开发区通过评审。我市被命名为省级文明城市、生态园林城市,宜君县荣获全省县城建设先进县。309省道川口至陈炉公路路基贯通，华夏南道、华原西道建成通车，改善农村公路241公里。在全省率先完成“黄标车”淘汰任务和清洁能源公交车全覆盖，铜川交通“一卡通”系统建成运行。新增天然气用户1.03万户，城市集中供热面积达到543万平方米。黄堡省级重点示范镇和照金、陈炉省级文化旅游名镇建设完成投资11.5亿元，超额完成年度任务。实施了美丽乡村示范片区5个、创建村80个。环境保护力度持续加大，拆除燃煤锅炉516台，全年收获优良天数242天，同比增加32天。全面推行河长制，完成市污水处理厂、新耀污水处理厂提标改造，出市断面水质基本达标。我市被列入国家山水林田湖生态保护修复项目示范区，开工项目18个，完成投资16.45亿元。全年植树造林12.6万亩，治理水土流失213.35平方公里。

　　（六）保障改善民生，共建共享促和谐。民生支出占财政总支出的81.5%，较上年提高1.37个百分点。城镇新增就业1.02万人，城镇登记失业率3.23%，重点群体就业得到较好保障，省级创业型城市创建工作通过验收。普惠性幼儿园占比达到83.17%，提前三年实现义务教育均衡发展和“双高双普”目标，在全省率先实现农村中小学教师周转房全覆盖。医药卫生体制改革、城市公立医院综合改革和医养结合、社会办中医改革试点持续推进，北京中医药大学第七临床医学院暨孙思邈医院运行良好。实现基本医疗保险跨省异地就医直接结算，基本医疗保险和大病医疗保险一站式办理，铜川矿业公司5.1万名职工医疗保险纳入地方管理，在全国地级市中率先建立全民意外伤害保险制度。国家公共文化服务体系示范区创建工作扎实推进，全民健身馆建成开放，文体惠民活动广泛开展。安全生产形势总体平稳，死亡人数、直接经济损失分别下降35.1%、94.5%，未发生较大及以上安全事故。印台区创建省级食品安全示范区通过验收。信访工作年度考核名列全省首位。持续开展“大巡防”工作，立体化社会治安防控效能不断提升，宜君县被授予全国平安建设先进县和信访工作“三无县”。我市入选2017中国最具幸福感城市。

　　各位代表，2017年是本届政府履职施政的开局之年，按照全面从严治党的要求，全面加强政府自身建设。认真学习贯彻党的十九大精神，常态化制度化推进“两学一做”学习教育。修订和严格执行《市政府党组议事规则》《市政府工作规则》，做实依法科学民主施政的制度保障。强化经济工作协调，坚持月例会、季考核制度。认真履行“一岗双责”，深入推进党风廉政建设，市本级“三公”经费支出同比下降7.6%，风清气正的政治生态得到巩固和发展。

　　这些成绩来之不易，是省委、省政府坚强领导、关心支持的结果，是市委统揽全局、科学决策的结果，是市人大、市政协及社会各界监督支持的结果，也是全市人民多年攻坚克难、久久为功成效累积的结果。在此，我代表市人民政府，向全市人民，向各民主党派、各人民团体和关心支持铜川发展的各界人士，表示崇高的敬意和衷心的感谢！

　　同时，我们也清醒认识到，铜川转型发展正处于爬坡过坎的关键阶段，发展不平衡不充分的问题仍然很突出。经济增长内生动力还不足，发展质量和效益还不高，实体经济、民营经济和县域经济发展滞后；城乡规划、建设和管理水平有待提高，生态环境约束趋紧，污染防治任务繁重；民生领域还有不少短板，脱贫攻坚任务艰巨，劳动就业、社会保障、文化教育、医疗卫生等领域仍有群众不满意的地方；政府职能转变还需加快，发展环境还不尽人意，一些政府工作人员的思想观念、责任担当、创新意识、本领能力与新时代新使命新要求还有差距。我们必须直面这些困难和问题，采取更加有效的举措，切实加以解决。

　　二、2018年的工作思路和预期目标

　　2018年是全面贯彻落实党的十九大精神开局之年，是改革开放40周年，是决胜全面建成小康社会、实施“十三五”规划承上启下的关键一年，也是铜川建市60周年，做好各项工作意义重大。党的十九大对新时代中国特色社会主义发展作出战略部署，新思想引领新时代，新目标开启新征程。省委十三届二次全会和省十三届人大一次会议，为认真践行“五个扎实”要求，全面落实“五新”战略任务，奋力谱写新时代追赶超越新篇章，指明了方向和路径。市委十二届三次全会对我市践行新思想、落实新战略、履行新使命，奋力追赶超越、加快转型发展，提出了“铜川行动方案”。时代是出卷人，我们是答卷人，人民是阅卷人。站在新时代历史方位，我们要紧紧围绕省市委的决策部署和目标定位，苦干实干、大干快干，从打基础利长远的事情干起，从群众最关心最期盼的工作抓起，从发展不平衡不充分的地方做起，聚精会神谋发展，心无旁骛抓落实，尽心竭力满足人民日益增长的美好生活需要，决不辜负这个伟大的新时代，决不辜负铜川人民的期盼，决不辜负组织的信任！

　　今年政府工作的总体思路是：以习近平新时代中国特色社会主义思想为指导，认真贯彻落实党的十九大、中央经济工作会议、十三届全国人大一次会议和省委十三届二次全会、省十三届人大一次会议、市委十二届三次全会精神，以建设西部传统产业转型升级示范城市为目标，坚持和加强党的领导，坚持稳中求进工作总基调，坚持新发展理念，紧扣社会主要矛盾变化，按照高质量发展的要求，全面落实“五个扎实”和“五新”战略任务，以供给侧结构性改革为主线，以追赶超越、转型发展为主题，统筹做好稳增长、促改革、调结构、惠民生、防风险各项工作，打好防范化解重大风险、精准脱贫、污染防治攻坚战，推动质量变革、效率变革、动力变革，促进经济社会持续健康发展。

　　今年经济社会发展主要预期目标是：生产总值增长7.5%左右，规模以上工业增加值增长6.6%左右，固定资产投资增长10%左右，社会消费品零售总额增长13%左右，地方财政收入增长8.5%左右，城镇登记失业率控制在4%以内，CPI涨幅控制在2.5%左右，城乡居民人均可支配收入分别增长8%和8.5%左右，单位生产总值能耗、主要污染物减排完成省上下达的指标任务。上述主要预期目标安排，充分考虑与“十三五”规划相衔接，尊重经济规律，体现远近结合和高质量发展的要求，确保经济运行在合理区间。做好今年的经济工作，必须牢牢把握好五个关键。

　　一是紧扣高质量发展新特征，推动质量变革、效率变革、动力变革。由高速增长转向高质量发展，是新时代经济发展的显著特征。必须深入践行新发展理念，认真落实“五个扎实”要求和“五新”战略任务，坚持质量第一、效益优先，着力提高全要素生产率，切实破解发展不平衡不充分问题，加快构建具有新时代特征、铜川特色的现代化经济体系。

　　二是紧扣追赶超越新目标，突出做好“三抓一稳一增”重点工作。追赶超越是习近平总书记对陕西的精准定位，也是我市转型发展的实现路径。必须抓重点、补短板、强弱项，把抓投资作为重要抓手、抓结构作为重大战略、抓环境作为必由之路、稳增长作为基本前提、增动能作为内在要求，加快实现经济总量追赶、质量效益超越、发展模式创新。

　　三是紧扣铜川转型发展新阶段，增强全面、协调和可持续发展能力。建成西部传统产业转型升级示范城市，是省第十三次党代会对我市的目标定位。必须抓住省上深化“一市一策”、支持铜川等资源型城市转型升级发展的机遇，坚持从实际出发，大胆创新，勇于实践，加快推进产业转型、城市转型、社会转型、体制机制转型，不断开创新时代转型铜川、美丽铜川、健康铜川、富裕铜川新局面。

　　四是紧扣社会主要矛盾新变化，不断提升保障和改善民生水平。人民群众对美好生活的向往就是我们的奋斗目标。必须坚持以人民为中心的发展思想，持续加大保障和改善民生工作力度，在推动高质量发展中加快补齐民生短板，创新社会治理，促进社会公平正义，使全市人民的获得感、幸福感、安全感更加充实，更有保障，更可持续。

　　五是紧扣新时代从严从实新要求，在实干快干中谱写发展新篇章。按照信念过硬、政治过硬、责任过硬、能力过硬、作风过硬的要求，全面提高履职本领，善于改革创新，勇于攻坚克难，一以贯之抓落实，一张蓝图干到底。强化督查考核问责，建立健全绩效评价、政绩考核体系，努力创造人民认可、经得起历史检验的新业绩。

　　三、2018年主要工作任务

　　（一）落实“五新”战略任务，竭力扩大有效投资。

　　把落实“五新”战略任务的着力点放在项目建设上，坚持目标导向和问题导向，优化要素配置，全力抓好项目建设和招商引资工作，建设具有铜川特色的现代化经济体系。

　　全力推进项目建设。围绕新型能源、新型建材、航空汽车材料、优质农产品生产物流、健康养生五大基地建设，扎实做好项目策划推进工作，全年策划项目投资1000亿元以上。实施市级重点项目200个，总投资1532亿元，完成投资237亿元，大秦铝业2万吨铝锂合金熔铸生产线、耀州窑文化基地企业孵化园等71个续建项目达到序时进度，陕建集团新型建材生产线、照金干部学院等30个项目建成投运，西延高铁铜川段、国家光伏发电技术领跑基地等69个项目开工建设。完善服务重点项目建设实施办法，坚持市级领导包联、驻工地专员包抓、项目集中开工、项目观摩等制度，力促重大项目建设实现新突破。

　　切实做好要素保障。成立项目要素保障领导小组，切实抓好项目征地拆迁、手续办理、环境优化和协调服务等工作。深化政银企合作，实现政府性融资担保机构业务县域全覆盖，积极推进普惠金融，提高金融服务实体经济的能力和效率。出台扶持激励政策，促进企业在多层次资本市场直接融资，支持企业上市发展。鼓励民间资本参与政府和社会资本合作（PPP）项目，激发民间投资活力。加大闲置、低效土地清理力度，做好重点项目用地保障。强化财政资金绩效管理，严控政府债务规模。优化金融生态环境，打击非法集资，有效防范金融风险。

　　狠抓招商引资工作。加大“走出去、请进来”力度，按照产业转型的思路，开展精准招商、全员招商活动，重“量”更重“质”，重“资”更重“才”，争取在招大引强、招才引智上有新突破。组织好第三届丝博会、第八届陕粤港澳经济合作活动周等招商活动，展现铜川新动能、新经济、新形象。增强“钉钉子”精神和“店小二”意识，强化考核抓推进，提高招商引资项目落地率和资金到位率。全年实际利用市域外资金200亿元以上。

　　（二）加快工业转型升级，增强持续发展动能。

　　以建设西部传统产业转型升级示范城市为目标，走绿色低碳循环发展之路，改造提升传统产业，培育壮大新兴产业，不断厚植转型发展新优势。

　　改造提升传统产业。按照“强铝、精煤、扩电、优水泥、兴陶瓷”的思路，抓好传统产业优化升级。以做优做强铝产业链为重点，大力发展高端铝型材、机电用铝合金等产品，提高为省内外航空航天、汽车工业、建筑型材、机电型材等产品配套率，打造全省航空汽车材料工业基地。制定促进煤化工、洗选业发展的政策措施，提升煤炭产业发展。积极争取增发电指标，扩大电力直接交易试点。鼓励发展低碳水泥，大力发展装配式建筑产业，创建省级装配式建筑试点示范城市。发挥耀瓷品牌优势，加快现代陶瓷产业发展。

　　加快新兴产业发展。实施创新驱动战略，围绕“中国制造2025”及陕西实施意见，推动互联网、大数据、人工智能和实体经济融合发展。加快产业技术研究院建设，构建以实体经济为主的产学研协同创新体系。积极实施光伏发电、风力发电等项目，推动绿色能源产业发展。以高低压输配电、光伏装备、风电装备、电线电缆等为重点，大力发展电力装备产业。以新区南部产业园、周陵农产品加工产业园为承载，加强政策指导和支持，促进食品产业发展。依托药王文化品牌，引进实施中医药产业化项目，促进大健康产业发展。持续推进质量强市建设，深入开展质量提升行动，大力弘扬劳模精神和工匠精神，加大品牌培育保护力度，提高产品、工程和服务的质量水平。

　　狠抓产业园区建设。加快推进董家河循环经济产业园、新区南部产业园汽车零部件产业发展，促进美鑫五大产业集群项目尽快建成投产。充分发挥铜川新材料产业园的区位、能源优势，以电线电缆、装配式建筑、新型建材等为重点，推动产业集群化发展。用好“飞地经济”政策，加快建设“飞地园区”，启动建设盐铜经济合作园。支持惠塬、王家河、陈炉双碑、彭镇等县域工业园区建设。完善园区基础设施，新建和改造标准化厂房不少于70万平方米，园区工业增速高于区县2-3个百分点。全市新增规模以上工业企业不低于20户，工业技改投资增长15%以上。

　　（三）实施乡村振兴战略，推进农业农村现代化。

　　按照产业兴旺、生态宜居、乡风文明、治理有效、生活富裕的总要求，深入推进农业供给侧结构性改革，着力构建现代农业产业体系、生产体系、经营体系，促进农村一二三产业融合发展，推动农业提质增效、农村文明进步、农民增收致富。

　　打好脱贫攻坚战。扎实开展“不忘初心、牢记使命，冲在一线、干在实处，帮贫解困促发展”大调研，深入实施贫困村提升工程和特殊困难群体帮扶行动，建立健全稳定脱贫长效机制。注重扶贫同扶志、扶智相结合，激发脱贫内生动力。坚持以产业扶贫为重点，强化“五个三”工作思路，实施贫困村特色产业推进行动。完成2031户6120人易地扶贫搬迁。探索建立“五位一体”金融扶贫模式。抓好7个深度贫困村脱贫攻坚工作。深化盐铜扶贫协作和经济合作。实现1.16万贫困人口脱贫、78个贫困村退出，宜君县达到脱贫摘帽标准。

　　发展特色现代农业。坚持质量兴农、绿色发展，推进农业转型升级。落实科技增粮措施，做好粮食生产功能区划定、建设、监管等工作，粮食总产达到20万吨以上。启动9个生态循环农业示范区创建和19个有机绿色农产品示范基地建设，高标准抓好耀州区国家级田园综合体试点项目。创建优质水果标准化示范园2000亩，改造低产园1万亩，加大铜川果品区域公用品牌宣传推介力度，力争将“铜川大樱桃”创建成中国驰名商标。支持食用菌、中药材、苗木花卉等产业发展。促进生猪、奶牛、肉羊、蛋鸡等畜牧业规模化、标准化发展。积极开发观光农业、游憩休闲、生态教育等服务。加快农业大数据应用，启动农业标准化生产、农产品质量安全监管、优势特色农产品展示平台“三网合一”工作。

　　深化农村综合改革。完成农村土地承包经营权确权登记颁证工作，落实第二轮土地承包到期后再延长30年政策。做好农村集体资产清产核资，抓好“三变”改革示范村建设。建立完善区县农村产权交易中心，稳步推进农村土地流转。完善农业社会化服务体系，实施新型农业经营主体、新型职业农民、农业专业技术人才培育工程，促进小农户和现代农业发展有机衔接，提高农业组织化程度和抗风险能力。

　　加快美丽宜居乡村建设。实施通村组沥青（水泥）路、安全饮水巩固提升和自然村通动力电“三提升”工程，加大生命安全防护工程实施力度，建成通村组沥青（水泥）路300公里，解决3万农村居民饮水安全问题。编制农村人居环境整治三年行动方案，加强农村面源污染防治，逐步完善农村垃圾处理、污水处理设施，实施病死畜禽无害化处理试点和化肥替代行动。开展“美丽乡村、文明家园”创建活动，加快改善农村人居环境，创建5个美丽乡村示范片区和83个美丽宜居示范村。

　　（四）坚持以全域旅游为引领，加快现代服务业发展。

　　以推进全域旅游示范市创建为抓手，加快旅游业全面提升，推进旅游业与各产业融合发展，推动服务业总量扩大、结构优化、业态创新。

　　促进全域旅游发展。把发展全域旅游、建设全景铜川作为转型发展的重要抓手，加快实施景区标准化建设、旅游民宿建设、旅游厕所建设、智慧旅游建设和文化遗产保护提升五大工程。实施旅游重点项目33个，完成投资20亿元以上。对标抓好国家中医药健康旅游示范市、照金5A级景区创建工作。扎实做好药王山、玉华宫、陈炉古镇的保护与发展。加快耀州国家全域旅游示范区、宜君省级旅游示范县创建工作，抓好耀州窑文化基地耀瓷小镇、王石凹工业遗址项目建设，提升秦人村落、申河湿地公园、溪山胜境、孔雀谷、花溪谷等景点品质。全年游客接待量、旅游总收入分别增长15%和20%以上。

　　培育壮大现代服务业。出台促进现代服务业发展的实施意见，研究制定服务业发展指导目录。建成陕西现代果业物流园果品配送中心和铜川鼎铭物流园，加快建设西北冷链物流园，开工建设农资产业园。加快推进中国西部工业品数字港、大唐跨境电子商务国际产业孵化园建设，建成国家水泥质量监督检验（铜川）中心。

　　促进消费供给升级。大力发展数字经济、分享经济、创意经济等新经济，支持服务外包、家居服务等产业，以新业态新模式激发新消费。加强与北京中医药大学、中国中医药科技开发交流中心等合作，加快中医药创新创业公共服务平台建设，大力发展中医药健康养生保健产业，推进康养城市建设。完善城乡流通网络设施，释放城乡消费潜力。

　　（五）深化供给侧结构性改革，促进实体经济发展。

　　坚持把发展经济的着力点放在实体经济上，深化要素市场化配置改革，重点在“破、立、降”上下功夫，用好国内国际两个市场、两种资源，拓展发展空间。

　　持续推进“三去一降一补”。严格执行环保、质量、安全等相关法规和标准，用市场化法治化手段推进“去产能”。在做好职工分流安置的前提下，有序推进市属“僵尸企业”处置工作。积极实施棚户区改造，加快建立多主体供应、多渠道保障、租购并举的住房制度。继续开展企业降本增效行动，清理涉企收费，降低用能、物流和制度性交易成本。加快剥离国有企业办社会职能，妥善解决历史遗留问题。推进国有资本授权经营体制改革，抓好企业公司制改革和整合重组。

　　深化“放管服”改革。开展营商环境提升年活动，抓好优化提升营商环境十大行动。全面实行“多证合一、一照一码”，最大限度利企便民。大力推行“互联网＋政务服务”，实施网上经办服务，推进“不见面审批”、群众办事“最多跑一次”。深化市场秩序整顿，加快信用铜川建设，完善守信联合激励和失信联合惩戒制度。认真落实支持民营经济发展的政策措施，加强各项产权保护，构建亲清新型政商关系，营造尊重、激励和保护企业家干事创业的社会氛围。全年新增私营企业300户以上。

　　加快对外开放步伐。主动融入陕西自贸区建设，加快形成内外联动、承东启西、连接南北的全面开放新格局。搭建产业、资源、信息、数据共享平台，借力发展枢纽经济、门户经济、流动经济，支持办好“一带一路”耀州窑国际学术研讨会和国际陶瓷文化展。实施外贸培育工程，扩大苹果、陶瓷、中药材等地产品出口，外贸进出口总额3.9亿元。

　　（六）深入推进新型城镇化，推动城乡区域协调发展。

　　全方位对接关中平原城市群发展规划，主动融入以大西安为核心的关中协同创新发展，坚持以人为本，持续抓好城乡规划、建设和管理，提高新型城镇化质量。

　　强化城乡统筹规划。推进多规合一，发挥规划的战略引领和刚性管控作用。完成新一轮城市总体规划修编工作，统筹资源、空间、产业布局，促进新耀协同创新发展、南北市区融合发展、宜君秀美山城建设。深化编制有关专项规划，实现城市控制性详细规划、乡村规划全覆盖。积极开展城市设计，优化空间形态，提升城市品质，让市民生活更美好。加强规划实施全过程监督，依法严查各类违法违规行为。

　　加强城乡建设管理。加快推进智慧城市、海绵城市和地下综合管廊建设，建成数字城管平台。完善新区基础设施，做好北市区基础设施改造。加快城市集中供热工程建设，做好供水、供气、排水老旧管网改造，推进公共充电桩建设。以国卫巩固提升为抓手，加强城市管理综合执法，探索建立街长制，在精治、共治、法治上下功夫。推进城镇、农村“厕所革命”，不断提升人民群众生活品质。完善非机动车道、城市步道、无障碍设施，做好综合市场、公共停车场的规划建设。用好城镇建设专项资金，加快推进黄堡重点示范镇和陈炉、照金文化旅游名镇及2个省上跟踪考核重点镇建设，推动产业、人口向城镇聚集，促进农业转移人口就地就近市民化。

　　加快县域经济发展。认真落实加快县域经济发展的意见，市、区县联动，加快提升县域综合实力。支持新区省级高新技术产业开发区建设，加大科技型企业培育力度，着力打造全市创新发展示范区。推动耀州区“四化”同步发展，做大做强优势主导产业，着力打造全市转型发展先行区。加快王益区休闲购物、配套加工、特色农业发展，推进三产融合发展。推动印台区产业项目建设，突破工业瓶颈制约，加快产业转型升级。发挥宜君县绿色生态优势，大力发展生态农业、避暑休闲产业，着力打造绿色休闲度假养生基地。

　　（七）加强生态文明建设，加快打造美丽铜川。

　　树牢绿水青山就是金山银山理念，全力打好污染防治攻坚战和蓝天保卫战，系统推进生态环境保护修复工程，让全市人民切实感受环境改善的成果，享受绿色福利。

　　着力解决环保突出问题。制定打好污染防治攻坚战方案，坚持刚性治标与系统治本并重，深入推进大气、水、土壤污染防治。启动新一轮大气污染防治三年行动，强化网格化监管，确保全年优良天数不少于261天。启动漆水河、沮河水质提升和生态补水项目，实施市污水处理厂污泥资源化处理工程，完成我市渭河流域综合整治三年行动目标。加快土壤污染源详查，搞好污染土壤管控、治理和修复试点。加强固体废弃物和垃圾无害化处置，推进资源化利用。开展第二次全国污染源普查工作。

　　加大生态保护修复力度。划定并严守生态保护红线，加快实施山水林田湖生态保护修复工程，做好矿山地质环境恢复治理。抓好国家森林城市、国家园林城市创建工作，坚持山上治本、身边增绿，继续实施城市、道路、流域、园区、景区、庭院重点区域造林绿化工程，全年造林10万亩以上。全面落实河长制、湖长制，加快赵氏河、沮河、福地湖国家湿地公园建设和试点，强化湿地保护和恢复。全年治理水土流失150平方公里。

　　完善环保监管体制。坚持“党政同责、一岗双责”，实行领导干部自然资源资产离任审计，全面落实自然资源资产管理和生态环境保护责任。健全环保督察、信用评价、信息强制性披露等制度，强化源头严控、过程严管、违法严惩，抓好中央和省委环保督察反馈问题整改，保持对环境违法行为的高压态势。

　　（八）切实保障和改善民生，持续增进群众福祉。

　　以造福人民为最大政绩，持续加大民生投入力度，倾心竭力多办利民实事、多解民生难事,兜牢民生底线，让全市人民生活一年更比一年好。

　　抓好创业就业工作。加大政策性金融支持力度，加快“双创”服务体系建设，促进高校毕业生、贫困劳动力、返乡农民工等重点群体就业创业。创建国家级创业孵化示范基地。全年发放创业担保贷款1.5亿元，城镇新增就业8000人以上。重视人才队伍建设，筹建铜川发展智库，制定招才引智政策措施，着力引进高端紧缺人才，加快产学研用一体化进程，全面优化人才结构。拓宽居民劳动收入和财产性收入渠道，不断提高收入水平。

　　完善社会保障体系。保持社会保险参保全覆盖，启动第三代社保卡发行工作。推进机关事业单位基本养老保险制度改革，继续提高退休人员基本养老金和城乡居民基础养老金。完善统一的城乡医疗保险政策，深化基本医疗保险支付方式改革，对市属国有困难企业退休人员基本医疗保险财政补助政策再延续4年。建成4个城市社区服务站、8个农村社区服务中心和4个社区老年人日间照料中心、50个农村幸福院，完成市老年服务中心一期主体工程。做好农村五保户、城市“三无”人员、高龄和失能老人等生活保障。重视残疾人事业，加强残疾康复服务。做好农村留守儿童和妇女、老年人关爱保护工作。支持、引导志愿服务和慈善事业健康发展。

　　办好人民满意教育。优先发展教育事业，落实立德树人根本任务。推进第三期学前教育行动计划，新建和提升改造公办幼儿园8所。推进义务教育优质均衡发展，实施薄弱学校改造，加快新区中小学校建设，着力解决“择校热”“大班额”等突出问题。实施高中攻坚计划，完成宜君县高级中学省级标准化高中创建任务，支持王益区优化整合高中教育资源，积极创建省级示范高中。全面提高职业教育基础能力，加快推进职业教育现代化。鼓励社会力量兴办教育，促进民办教育健康发展。聚焦高素质专业化，加强师资队伍和师德师风建设。

　　提升健康服务水平。全面实施《“健康铜川2030”规划纲要》，推动更高水平的全民健康。深化医药卫生体制改革，扩大公立医院改革成效。启动实施新一轮改善医疗服务三年行动计划，完善分级诊疗制度，发挥医疗联合体作用。抓好市人民医院、矿务局中心医院、北京中医药大学第七临床医学院暨孙思邈医院“三甲”医院创建工作。在全省率先实现危重孕产妇及新生儿救治中心全覆盖。加强村卫生室和社区医疗机构建设，提升基层医疗服务能力和质量。

　　加强公共文化建设。大力弘扬和践行社会主义核心价值观，传承优秀传统文化，加强“厚德陕西?德润铜川”宣传教育，讲好“铜川故事”，巩固提升省级文明城市创建成果。加快铜川大剧院、工人文化宫、博物馆、体育馆建设，抓好国家公共文化服务体系示范区建设，打造铜川特色文化品牌。广泛开展全民健身活动，全力备战第十六届省运会。完善政策、深化改革、健全机制，加快壮大文化产业。办好铜川建市60周年系列庆祝活动，大力弘扬铜煤文化和矿工精神，激发全市人民干事创业的热情。

　　推进社会治理创新。加强社会治理制度建设，打造共建共治共享的社会治理格局。强化安全生产红线意识，坚持主体责任与监督责任同步到位、管理能力与技术防范同步加强、重点领域与面上工作同步推进、党委与政府同步发力，大力提升安全生产整体水平，坚决遏制重特大安全事故发生。加强食品药品监管，守护好群众舌尖上的安全。加强应急基础建设，提升防灾减灾救灾能力。全面实施“七五”普法规划，深入落实“谁执法谁普法”的普法责任制。持续推进“雪亮工程”建设，完善立体化社会治安防控体系，严厉打击各类刑事犯罪，依法开展扫黑除恶专项斗争。加强重大决策社会稳定风险评估，依法化解各类社会矛盾和风险隐患。强化城乡社区治理，推进“四社联动”，做好第10次村委会换届选举工作。加强和规范住宅小区物业管理，提升物业管理服务质量。

　　四、奋力开创政府自身建设新局面

　　新时代要有新气象，更要有新作为。落实全面从严治党要求，以永远在路上的执着全面加强政府自身建设，提高政治站位，转变政府职能，努力建设人民满意的法治政府、廉洁政府和服务型政府。

　　提升履职水平。按照抓首要、大学习、促发展的要求，深入学习贯彻习近平新时代中国特色社会主义思想和党的十九大精神，常态化制度化推进“两学一做”学习教育，扎实开展“不忘初心、牢记使命”主题教育，切实增强“四个意识”，坚定“四个自信”，始终与以习近平同志为核心的党中央保持高度一致。强化公职人员学习培训，大兴调查研究之风，切实做到“五个过硬”，着力增强“八种本领”，不断提高适应新时代、实现新目标、落实新部署的能力。

　　严格依法行政。坚决维护宪法权威，尊崇法治、敬畏法律、依法办事，把政府活动全部纳入法治轨道，严格规范公正文明执法。深化机构改革，优化政府机构设置和职能配置。健全政府法律顾问制度，规范行政复议、行政应诉，积极开展法治政府示范创建活动。自觉服从市委的领导，依法接受市人大及其常委会的监督，自觉接受人民政协的民主监督，主动接受社会和舆论监督。认真听取人大代表、政协委员、民主党派、工商联、无党派人士和各人民团体的意见，办好人大代表建议和政协提案。

　　扩大政务公开。推进“阳光政府”建设，拓宽政府信息公开范围，开展部门区县政务公开第三方评估。坚持公民代表走进政府、大学生到机关见习、公众代表列席市政府重要会议等制度，畅通群众与政府互动渠道。凡涉及公众利益的重大事项，必须广泛征求各方面意见，做到科学民主依法决策。举办市政府政策例行吹风会，建立发布、解读、回应“三位一体”政务公开新格局，保障群众的知情权、参与权、表达权和监督权。

　　强化正风肃纪。认真落实《市委常委会贯彻落实中央八项规定实施细则的实施办法》，驰而不息整治“四风”，切实纠正各种隐形变异问题。全力支持纪委、监委工作，深化标本兼治，坚持无禁区、全覆盖、零容忍惩治腐败，彻底肃清冯新柱流毒影响，努力营造风清气正的政治生态。持续精简会议和文件，压缩一般性支出，确保“三公”经费只减不增。用好“三项机制”，为积极干事者撑腰鼓劲，对庸政懒政者严肃问责，以实际行动赢得人民群众的信任和支持。

　　各位代表，新时代赋予新使命，新征程呼唤新担当。让我们紧密团结在以习近平同志为核心的党中央周围，以习近平新时代中国特色社会主义思想为指导，不忘初心、牢记使命，勠力同心、砥砺奋进，为决胜全面建成小康社会、开启新时代“四个铜川”建设新局面而努力奋斗！
